# Local Housing Characteristics Associated with Early Childhood Development Outcomes in Australian Disadvantaged Communities

**DOI:** 10.3390/ijerph16101719

**Published:** 2019-05-16

**Authors:** Karen Villanueva, Hannah Badland, Robert Tanton, Ilan Katz, Sally Brinkman, Ju-Lin Lee, Geoffrey Woolcock, Billie Giles-Corti, Sharon Goldfeld

**Affiliations:** 1 Murdoch Children’s Research Institute, 50 Flemington Road, Parkville, VIC 3052, Australia; julin.lee@mcri.edu.au (J.-L.L.); sharon.goldfeld@rch.org.au (S.G.); 2 Centre for Urban Research, Royal Melbourne Institute of Technology (RMIT) University, Melbourne, VIC 3000, Australia; hannah.badland@rmit.edu.au (H.B.); billie.giles-corti@rmit.edu.au (B.G.-C.); 3 Centre for Community Child Health, Royal Children’s Hospital, 50 Flemington Road, Parkville, VIC 3052, Australia; 4 National Centre for Social and Economic Modelling (NATSEM), University of Canberra, Canberra, ACT 2601, Australia; robert.tanton@canberra.edu.au; 5 Social Policy Research Centre, the University of New South Wales, Kensington, NSW 2052, Australia; ilan.katz@unsw.edu.au; 6 Fraser Mustard Centre, Telethon Kids Institute, Adelaide, SA 5000, Australia; sallyb@ichr.uwa.edu.au; 7 School of Population Health, The University of Adelaide, Adelaide, SA 5000, Australia; 8 University of Southern Queensland, Darling Heights, QLD 4350, Australia; Geoffrey.Woolcock@usq.edu.au; 9 Department of Paediatrics, University of Melbourne, Parkville, VIC 3052, Australia

**Keywords:** urban planning, neighbourhood, community, early childhood development, family, mixed methods, inequity, housing

## Abstract

Disadvantaged communities tend to have poorer early childhood development outcomes. Access to safe, secure, and stable housing is a well-known social determinant of health but there is a need to examine key features of neighbourhood housing that reduce early childhood development inequities. The 2012 Australian Early Development Census (AEDC), a population-wide measure of early childhood development, and the Australian Bureau of Statistics Socio-economic Index for Areas Index of Relative Socio-economic Disadvantage were used to select fourteen disadvantaged local communities in five Australian states and territories based on those performing better (off-diagonal), or as expected (on-diagonal) on the AEDC relative to their socio-economic profile. Between 2015–2017, qualitative and quantitative housing data were collected in the local communities. In total, 87 interviews with stakeholders, 30 focus groups with local service providers and parents, and Australian Census dwelling information were analysed. A comparative case study approach was used to examine differences in housing characteristics (e.g., public housing, density, affordability, and tenure) between disadvantaged local communities performing ‘better than expected’ and ‘as expected’ on early childhood development. Perceived better housing affordability, objectively measured housing tenure (ownership) and perceived and objectively measured lower-density public housing were housing characteristics that emerged as points of difference for disadvantaged local communities where children had relatively better early childhood development outcomes. These characteristics are potential modifiable and policy sensitive housing levers for reducing early childhood development inequities.

## 1. Introduction

Early childhood (0–8 years) is a time when environments contribute to the foundations for physical, social, emotional, and cognitive development [1]. Young children exposed to stimulating and positive environments (family, services, neighbourhood) develop foundational skills in learning, communication, problem-solving, and decision-making [2]. Conversely, young children exposed to negative environmental stress and instability are more likely to suffer poor developmental outcomes now and into the future [3].

The science supporting the ecology of childhood suggests that children develop in multiple contexts including the family, peer group, and broader social (e.g., social capital) and physical environments (e.g., local destinations, walkability) [4,5]. For young children, the home environment is typically their primary development context, yet their home sits within the wider neighbourhood context, of which children and families with young children spend a considerable amount of time [6,7]. Thus, the home and neighbourhood settings are consistently recognised as fundamental spheres of influence when taking an ecological perspective of early childhood development [4].

Housing is a well-recognised social determinant of health, and of interest for public health, social policy, urban design and planning [8,9]. Access to safe, secure and stable housing [10] has been linked to children’s developmental outcomes, such as language development [11], behavioural problems, and anxiety and depression [6,12]. The mechanism through which housing impacts child development can be direct or indirect [6]. For example, while poor quality of housing (e.g., mould growth, pest infestation, noise, overcrowding) can directly affect children’s physical health [7,13], it may also influence parent mental health [14]. A parent’s poor mental health (e.g., experiencing stress, anxiety, depression) may affect parenting practices and styles (e.g., punitive or harsh parenting), which in turn can negatively affect their children’s behaviours [15], cognitive development [16,17,18,19] and social development [20].

Despite the paucity of research, other studies have found access to affordable housing, housing stock, housing tenure, public housing, and housing density may influence early childhood outcomes [7,21]. These aspects of housing are thought to be important modifiable independent factors that conceptually fit within a social determinants approach to considering disadvantage and child outcomes [22]. Neighbourhood disadvantage has long been recognised as a risk factor for poor early childhood development [23,24]. Cross sectional data show that, in general, the more disadvantaged the neighbourhood, the poorer the health and wellbeing outcomes of the residents [25]. Addressing ‘locational’ disadvantage through modifiable social determinants of health (such as housing) is a priority if we are to reduce inequities in health and wellbeing [26,27]. Research shows that compared with more affluent areas, disadvantaged areas frequently have disproportionately poorer quality housing [28], services and destinations [29], and more public housing stock [30]. Other risk factors typically associated with more disadvantaged neighbourhoods (e.g., social issues such as crime and incivilities) also contribute to poorer child outcomes [28,31]. Moreover, family needs and resources (e.g., income), characteristics (e.g., family size and structure) and preferences influence the types of dwellings and neighbourhoods in which they reside. For families experiencing ‘double disadvantage’ (i.e., poor families living in poor neighbourhoods), these environmental effects on young children’s development is exacerbated [6,19]. Four characteristics of housing thought to be important for early child development are briefly summarised below.

### 1.1. Housing Affordability

Housing affordability has long been a research, policy, and citizen focus. Housing affordability is closely linked to the adverse effects of poverty [31] with housing stress usually considered when the housing cost burden (e.g., rent or mortgage repayments) is greater than 30% of gross income for those in the lowest 40% of equivalised disposable household income [32,33]. Housing affordability has been postulated to indirectly affect early childhood development through material and financial hardship, which may affect family stress. For example, rising housing costs may limit disposable income available to spend on other living costs, such as food, school activities and health care [34]. Housing affordability issues may force families to relocate neighbourhoods, which can also affect access to health, education and social services, and impact social connections [35]. Families experiencing economic hardship and employment difficulties can be problematic for children’s development and mental health [31].

### 1.2. Housing Tenure

Home ownership (those buying or owning their house vs renting) has been associated with less behavioural problems [36,37] and better educational outcomes in children [36]. While socio-economic status may influence home ownership status, home ownership may indirectly affect child development because it provides families with greater opportunity to maintain consistency and stability in daily routines, social interactions [38], and life experiences [36], as families who own homes are less likely to move compared with families who rent. Residential stability or less transience may facilitate feelings of neighbourhood attachment and satisfaction [39]. Indeed, home owners may have stronger neighbourhood attachment and a vested interest in their wider community. For example, Boyle (2002) suggests that home owners have stronger incentive to monitor and control problem behaviour of not only their own children, but in those of their neighbours [37]. For children, residential stability means fewer school transitions [31,36,40]. While the evidence is mixed, frequent school moves have generally been associated with declines in academic performance [41,42] and child internalising behaviour problems (e.g., withdrawn/depressed) [43].

### 1.3. Public Housing

For low-income earners, public housing represents a feasible housing option that supports housing affordability through rental subsidies and greater security of tenure (than private renters) [7,44]. Yet, public housing residents are more likely to be exposed to more crime, high unemployment, or other social issues within a concentrated area [45]. Aside from social issues, substandard housing may be more likely for those living in public housing developments [46]. The evidence on public housing and early childhood development appear mixed [7]. Structural housing quality, including physical condition, appearance and maintenance often influences how others (e.g., neighbours) view local residents [47]. As a result, residents may feel stigmatised by the larger community and may internalise other’s negative perceptions of them [18]. The stigma attached to those living in public housing and ‘bad’ neighbourhoods can influence self-esteem. Moreover, without provision of semipublic space and facilities around public housing, families may be more likely to stay indoors and experience minimal opportunity for developing informal social networks, social support, protection and informal social control compared to other disadvantaged neighbourhoods with these facilities [48].

### 1.4. Housing Density

Higher density living may negatively affect children’s development outcomes [49], but the findings are indicative rather than conclusive [50,51]. One pathway in which housing density may influence child outcomes is through perceived neighbourhood safety [52]. Living in unsafe environments (e.g., perceived crime, personal safety) may influence social interaction [53] and social networks [54], leading to feelings of social isolation, which has a negative influence on parent mental health [55]. Neighbourhood safety concerns can influence family practices and parent restrictions on children’s opportunities to play outside [56], and interact with others locally [57]. For example, Evans (2011) found mothers of young children living in high-rise developments expressed difficulties in monitoring children’s outdoor play because of crime and safety concerns [19]. Whitzman and Mizrachi (2012) found children living in high-rise housing were concerned about traffic volumes and few safe crossing points [58]. Others have found that children’s outdoor play and neighbourhoods with better social capital among residents encourage positive child development, even in poorer neighbourhoods [5,59,60,61]. Socially supportive neighbourhoods may encourage people to interact and ‘look out’ for each other, thereby preventing neighbourhood crime and other problem behaviours [62].

### 1.5. Study Rationale and Aim

A recent review of neighbourhood associations with early childhood development suggests the need for a deeper exploration of how children and their families interact with their neighbourhood [63]. In particular, how the variability in access to and the quality of neighbourhood attributes (e.g., housing) shapes child development and how it may contribute to developmental inequities [63]. Others have emphasised the need for more qualitative and mixed methods research to better understand the mechanisms and pathways in which housing affects people’s health [64,65].

This study contributes to this research gap; its aims are to describe and examine the pathways through which aspects of housing are related to early childhood development using mixed methods data from two types of disadvantaged local communities. Our comparative case study of housing issues compares disadvantaged communities with ‘as expected’ child development outcomes (i.e., poorer child development outcomes in a disadvantaged area) and disadvantaged communities with ‘better than expected’ child development outcomes (i.e., better child development outcomes in a disadvantaged area) [66] to create a counterfactual scenario that challenges the notion that child development outcomes are driven by socio-economic status. Through a range of community perspectives, we examine how selected housing characteristics (i.e., housing affordability, housing tenure, public housing and housing density) may affect early childhood development. Understanding which housing attributes make a difference to child development outcomes in disadvantaged areas could elicit potentially modifiable policy levers to promote early childhood development outcomes and thereby reduce inequity.

## 2. Materials and Methods

This paper utilised data from the Kids in Communities Study (KiCS), a mixed methods study conducted in urban and major regional areas across five Australian states and territories (Victoria, New South Wales, South Australia, Queensland and the Australian Capital Territory). KiCS aimed to investigate community-level factors influencing young children’s development in communities of varying disadvantage. Between 2015–2017, qualitative and quantitative methods were utilised to investigate community-level factors conceptualised within five community domains of influence (i.e., physical, service, social, socio-economic and governance environments). [67] The Melbourne Royal Children’s Hospital Human Research Ethics Committee provided ethics approval (#30016), and further ethics approvals were received from other states and territories as required [68]. Detailed information about the KiCS study is previously published [66,68].

### 2.1. Selection of Local Communities

**Figure 1:**
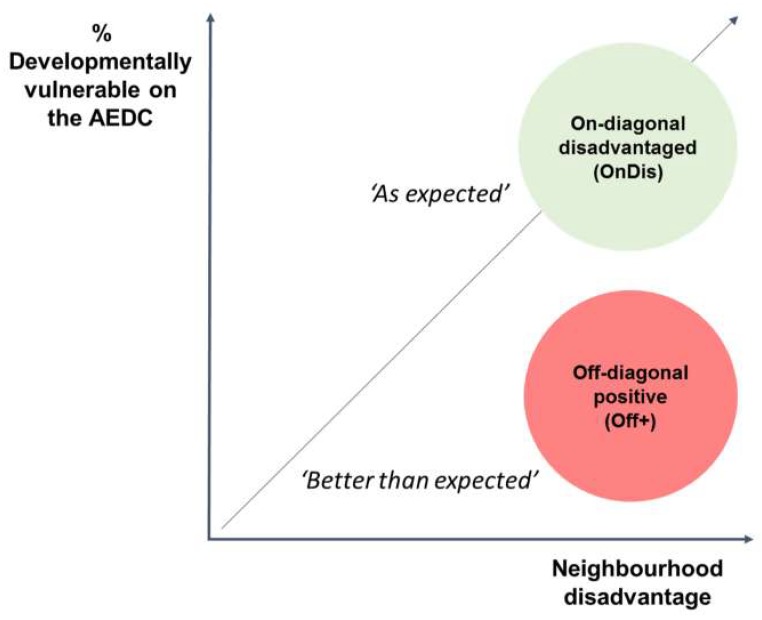
On- and off-diagonal local communities. Source: adapted from Goldfeld et al. 2018 [71]; AEDC: Australian Early Development Census; Developmentally vulnerable: % Developmentally vulnerable on at least one (of five) AEDC domains; Neighbourhood disadvantage: Australian Bureau of Statistics Socio-Economic Index for Areas—Index for Relative Socio-economic Disadvantage (IRSD).

To select local communities, KiCS utilised the 2012 Australian Early Development Census (AEDC), a population measure of early childhood development which assesses children in their first year of school across five developmental domains. The scores were used to classify children as developmentally vulnerable or on track [69]. Using a quintile-quintile matrix of Australian Bureau of Statistics (ABS) Socio-economic Index for Areas Index of Relative Socio-economic Disadvantage (SEIFA-IRSD) (area-level socioeconomic data) and AEDC data, disadvantaged local communities were identified. The KiCS case study approach compared community-level factors in neighbouring AEDC local communities classified as ‘on-diagonal disadvantaged’ (herein referred to as ‘as expected’) and ‘off-diagonal positive’ (herein referred to as ‘better than expected’), as determined by children’s aggregated AEDC scores relative to the local community’s disadvantage classification [66] (represented in Figure 1). An AEDC ‘local community’ [68] in urban and large regional areas equates to approximately 10,000 persons per area on average (i.e., a ‘suburb’) [70]. These local communities are clustered within larger AEDC ‘communities’ (municipalities or local government areas). Seven matched-disadvantaged community pairs were selected (i.e., each pair consists of one ‘as expected’ and one ‘better than expected’ local community), being one pair in Victoria, three in New South Wales, two in Queensland, and one in the Australian Capital Territory. Two pairs were in regional areas and five pairs were in major city areas [70].

### 2.2. Data Collection and Field Work

Table 1 describes the housing characteristics of interest in relation to early childhood development. To explore housing influences on families with young children, focus groups and interviews were conducted by trained research assistants between 2015–2017 using semistructured interview and focus group guides. Quantitative data included 2011 ABS Census dwelling information, and is included here for a descriptive snapshot of housing characteristics for the chosen communities [68].

#### 2.2.1. Qualitative Data

For each community, 8–15 semistructured interviews were conducted with a range of stakeholders (e.g., managers of early years’ services, local government staff, and school principals). Stakeholders were recruited through purposive and snowball sampling [72]. Interviews were conducted until the point of data saturation (i.e., no ‘new’ information obtained). While the interview questions did not focus specifically on housing issues, we asked open-ended questions about community factors they considered as positive and negative (challenges or difficulties) for young children and families. Interviews ranged from 35–90 min in duration. One stakeholder interviewee requested their recording to be omitted from transcription; however, they granted permission for notes to be coded.

For each local community, at least two focus groups were undertaken, one with local service providers for the early years (e.g., primary school teachers, maternal and child health practitioners, child care service providers) and one with parents of young children aged 0–8 years living in the local community. We aimed to recruit at least four people in each focus group. Where there were low numbers of focus group participants (e.g., <4 people), efforts were made to conduct another focus group for that community. Service providers and parents were recruited through stakeholder engagement. Parents were also recruited through distributing flyers through local organisations, and/or snowball sampling. Parents were reimbursed with an AUD$25 supermarket/department store gift card for their participation. Interviews using focus group questions were completed where a focus group could not be organised. Focus groups were held for 45–90 min with open-ended questions about each community domain, including housing. All focus groups and interviews were audio recorded (using a digital recorder and/or smart phone) and participants were asked to provide written consent for taking part. Data collection approaches varied by state/territory and community. Reasons for the variation included ethics rejections from government and Catholic education primary schools, and challenges in parent recruitment.

#### 2.2.2. Quantitative Data

Quantitative measures relevant to both early childhood development and housing were informed by earlier peer reviewed and grey literature reviews [73]. Although quantitative and qualitative data were originally collected concurrently, themes emerging from qualitative data analysis also helped inform the identification of further quantitative housing measures. That is, emerging qualitative themes informed whether an aligned (or ‘matching’) quantitative measure should be collected and analysed. Not all qualitative themes have a corresponding quantitative measure. ABS Census dwelling information (2011) was used to create objective measures of housing at the local community level, as described in Table 1 [74].

### 2.3. Analysis

In total, 87 interviews, 16 service provider focus groups (110 people), and 14 parent focus groups (79 people) were conducted for the sample of matched-disadvantaged community pairs. Interviews and focus group recordings were transcribed using an external transcribing service (Rev.com). Transcripts were quality checked by researchers and imported into QSR International’s NVivo v11, a software designed to assist with organisation and coding of qualitative data [76].

Thematic analysis using deductive and inductive processes was used to analyse housing characteristics. Due to the large amount of qualitative data and multiple coders (six researchers in total), a coding framework based on the KiCS conceptual framework (i.e., the five community domains) and previous literature was developed and refined by the KiCS’ research team [68]. Content analysis using a deductive approach was used to help ‘code’ the data using predefined categories (nodes) in NVivo. Information that did not fit within the existing codes but could be important for analysis was coded as ‘other useful information’. To ensure analytical rigour and consensus among the coders, issues were consolidated through regular team coding discussions and updating shared documentation; such approaches have been used in previous studies [77,78,79]. We applied an iterative categorisation (IC) technique to inductively summarise and analyse the coded content line-by-line in each node (category) [80]. The analysis techniques identified themes or factors which may facilitate or hinder early childhood development in the ‘better than expected’ and ‘as expected’ local communities.

To analyse each quantitative housing measure, we explored whether there was a ≥5% difference between the ‘better than expected’ and ‘as expected’ local community within each matched-disadvantaged community pair. We considered a ≥5% difference as a ‘large difference’, as the data were from a whole of population Census therefore accurate for small areas with no confidence intervals. To analyse both qualitative and quantitative data, we compared ‘better than expected’ and ‘as expected’ local communities within and across matched-disadvantaged pairs in the approach described below.

#### 2.3.1. Analysis within Matched Disadvantaged Community Pairs

We compared ‘better than expected’ and ‘as expected’ local communities *within* each matched-disadvantaged community pair. To do this, we analysed qualitative and quantitative housing characteristic data to elicit themes/factors that were different between the local communities, despite being both similarly disadvantaged at the neighbourhood level. We developed a hypothesis for each theme/factor based on previous literature (Table 2). For example, the proportion of public housing is less in ‘better than expected’ compared with ‘as expected’ local communities.

#### 2.3.2. Analysis across Matched Disadvantaged Community Pairs

To assist in reaching conclusions across the communities we considered a ‘consistent’ pattern existed when the majority (at least four of the seven) of matched-disadvantaged community pairs supported or refuted the hypothesis (Table 2).

## 3. Results

Table 2 presents findings of the qualitative and quantitative housing factors in relation to the stated hypotheses. The findings help identify housing factors (i.e., affordability, tenure, density and type) as potential reasons which might have led to the ‘better than expected’ early childhood development outcomes in local communities compared to their matched ‘as expected’ local communities. Our sample of only disadvantaged local communities intended to control for neighbourhood disadvantage and enabled the exploration for whether housing might provide a structural opportunity and resource to explain why children have better early childhood development outcomes despite living in a disadvantaged area. Our interpretation is based on consistent patterns differentiating the seven matched-disadvantaged community pairs (i.e., across ≥4 of the 7 matched-disadvantaged community pairs).

### 3.1. Housing Affordability

Housing was perceived as being more affordable in five of the seven ‘better than expected’ disadvantaged local communities compared with their ‘as expected’ counterparts. This was not supported by the objective measure of housing stress [31]. Housing was perceived as being more ‘desirable’ in four of the seven ‘better than expected’ local communities. However, housing affordability was also perceived as ‘more of an issue’ in five of the seven ‘better than expected’ local communities because of residential displacement of more disadvantaged families.

### 3.2. Housing Tenure

Compared with ‘as expected’ local communities, six of the seven ‘better than expected’ local communities had less private rentals and more home ownership, as identified through the quantitative data. While not consistently raised in most matched pairs, transience and stability were mentioned as important mechanisms in which housing tenure affected early childhood outcomes.

### 3.3. High-Rise Density Public Housing

Compared with the ‘better than expected’ local communities, five of the seven ‘as expected’ local communities had higher levels of public housing (both perceived and objectively-measured). Public housing type emerged as being important from the qualitative findings. Compared with ‘as expected’ local communities, five of the seven ‘better than expected’ local communities perceived more separate or semidetached lower density public housing being available, rather than ‘high-rise’ density housing types (e.g., apartments and townhouses). ‘As expected’ local communities perceived more high-rise density public housing. The objective data showed there was more high-rise density housing types (e.g., apartments and townhouses ≥3 storeys) present in the ‘as expected’ local communities, but this measure was not specific to public housing per se and included private housing.

## 4. Discussion

Our findings confirm that certain aspects of housing are important for early childhood development, especially for disadvantaged families and communities. Despite all areas being similarly socio-economically disadvantaged, more affordable housing, fewer renters and less high-rise high density public housing were three themes that strongly emerged. These may, at least in part, explain why some local communities had better early childhood development outcomes than others. In this study, other housing-related neighbourhood factors were raised, such as gentrification processes and neighbourhood stigma. There is no doubt that the way housing characteristics influence early childhood development is complex and involves interactions between sociodemographic factors, parent and family factors, and the wider neighbourhood. Indeed, housing characteristics also did not occur in isolation; factors such as housing tenure (owned, rented, publicly rented), type (higher-rise density vs. lower density housing), and cost (affordability) were interrelated [6]. While we were unable to comprehensively test the pathways in which housing influences early childhood development, it is clear that housing continues to be a particularly important issue for families living in disadvantaged communities. In discussing the results from the study there is an opportunity to consider how mixed methods can inform the conclusions.

Although affordable housing may be perceived as ‘positive’ by ‘better than expected’ local communities, it was also seen as a concern with negative impacts for subpopulations within the community. For example, participants perceived that affordable housing resulted in more issues for the most disadvantaged residents in the community; poorer families may be displaced as less disadvantaged residents move into the area to take advantage of the relatively more desirable and affordable housing. The findings suggest a trend of gentrification in the ‘better than expected’ local communities, a process of neighbourhood change that implies a changing residential class-income profile [35] (e.g., mixed income). Thus, local communities with ‘better than expected’ early childhood development outcomes may be slightly less disadvantaged than local communities with ‘as expected’ early childhood development outcomes. This is keeping in mind that the local communities are still disadvantaged, i.e., this is not comparing wealthy to poor communities.

Our research found that housing affordability is one of the main drivers of neighbourhood gentrification. *“People move here because it’s affordable”* (Focus group (FG) 47, parent, ‘better than expected’). As demand for housing increases and property values rise in gentrifying areas; those who are more disadvantaged may be forced to move away from their neighbourhoods (to areas where they can afford to live), social networks, and place of employment [81]. *“Our families may find that they may be able to afford slightly more for accommodation, because they’ve been used to paying more in [the City]. So, what happens is then a ripple effect, of our families who were used to paying for less, the competition changes, and they might be forced further out”. “That’s where you see that transient nature take over. People who can afford to pay for that from [the City] will come up and buy those, and the other people will be pushed farther out”* (FG32, parent, ‘better than expected’). Over time, locational disadvantage may become more pronounced in very disadvantaged areas (i.e., concentrated poverty), as those who cannot afford to leave are forced to remain in the neighbourhood.

In our study, there were less renters (and more home owners) in disadvantaged local communities where children were doing ‘better than expected’ compared with those doing ‘as expected’. It may not be home ownership per se, but a marker of housing stability [82]. Perceived secure and stable housing (either home ownership or rentals) was important in our qualitative data regardless of local community diagonality. Examples of the effects of transience or less stable housing was raised by participants as being impactful on young children’s ability to invest in meaningful relationships and friendships. *“Perhaps the children are not giving, not investing as much in relationships with their friendships and so on, because they’re passing through”* (FG04, service provider, ‘better than expected’). Higher rates of residential mobility and transience may influence social networks and sense of community [12]. For example, while people moved into the area because of perceived affordable housing, some were perceived as being *“stuck up”*. *“They go everywhere else for everything else [e.g., for daily needs] and don’t mix in with everyone”* (FG29, parent, ‘as expected’).

For some, gentrification was viewed positively; well-resourced disadvantaged areas may experience the benefits of gentrification. Gentrification can stimulate changes to the local area such as noticeable upward movements in social status, e.g., households characterised by higher incomes, university educations, and employment in professional positions [83]. There is some evidence that socio-economically diverse local communities are protective of early childhood development, compared with more homogenous suburbs with concentrated poverty [63]. That is, living near affluent neighbours appears to be protective of early childhood development compared with living near poor neighbours [84]. Participants mentioned *“It’s also how your neighbours live. They expect the handouts in [‘as expected’]. Whereas in [‘better than expected’] it’s not so condensed and confined. They can see how the other half live and they want to aspire to that”* (FG45, service provider, ‘better than expected’).

Improvements in local infrastructure and amenities (e.g., shops, public transport) [85] may appear within gentrifying areas, along with changes to the public image of the neighbourhood, and improvements in perceived neighbourhood safety [86]. Place-based and health studies have demonstrated associations of surrounding built environment features (e.g., quality destinations and services, availability of parks, traffic exposure) [87] and neighbourhood safety and crime [88] on children’s health behaviours and outcomes. Indeed, policy responses have included the creation of mixed-income developments by deconcentrating and dispersing poverty, and there is evidence to suggest that disadvantaged children living in these areas benefit not only from the exposure to more affluent residents but the presence of a wider variety of services and institutions that meet the needs of a more diverse population [89].

Less public housing (in both qualitative and quantitative data) was a differentiating characteristic between disadvantaged local communities doing ‘better than expected’ compared to disadvantaged communities that were ‘as expected’ in early childhood development outcomes. However, we found that it was not simply the mere presence (or absence) of public housing; rather, public housing type (e.g., detached single housing or higher-rise townhouses and units) and its distribution (e.g., located in concentrated pockets or otherwise ‘scattered’) made a difference. For example, participants referred to public housing as ‘not being so obvious’ (Interview (INT) 138, stakeholder, ‘better than expected’) if it was not higher-rise density housing types concentrated in the same area. *“One of the issues with public housing is, that it can be grouped together in say, small sizes in terms of inside their home and then only in the one spot. It’s a bit different if you’re in a house, with just one neighbour there, whereas [if] you’re in a group of a dozen or something and then a couple of people are a bit volatile or whatever. That affects everybody in a close area”* (INT070, stakeholder, ‘better than expected’).

*“…the condition of the department of housing houses appears to be better. In [‘as expected’] there’s a lot of very close-together tiny town houses and units. Less obvious in [‘better than expected’]—it’s more of a house and lot”* (INT125, stakeholder, ‘better than expected’). Such findings have implications for the design of public housing types and distribution (dispersion) in the community.

Public housing residents may feel stigmatised by the larger community and may internalise other’s negative perceptions of them [18]. Stigma attached to public housing and ‘bad’ neighbourhoods (concentrated poverty) may affect those living in stigmatised areas in a variety of ways, including job opportunities and self-esteem [90]. *“It’s the highest government housing area. It gets all those names, labels, and things attached”* (INT025, stakeholder, ‘as expected’) These negative assumptions seek to devalue or discredit marginal groups from full social acceptance [91,92], and can have a negative impact on children living in those areas [18]. Growing up in areas with a negative reputation may likely affect children’s self-esteem and aspirations for the future and were an emergent consistent theme in the communities (results not reported here). In addition to policies focusing on public housing type, dispersion and quality, finding ways to facilitate sense of community may help: *“There’s a ready supply of public housing for people in significant need so that’s a good thing. There’s still a sense of community within that area and certainly something that the schools and pre-schools reinforce either in their space or geographically nearby. I think that those within that space view it in a very positive way”* (INT082, stakeholder, ‘better than expected’).

Our findings highlight study strengths and limitations, and implications for policy and future research. In particular: (1) the importance for more policy-focused research on housing and neighbourhood level child health and developmental inequities; (2) more specific housing measures; and (3) future research that integrates qualitative methods with stronger quantitative studies to examine the pathways proposed in this paper. For example, gentrification and displacement from housing affordability, and high-rise density public housing were important themes in this study. Previous studies have also shown that mixed-income developments may benefit young children [35,84], and a systematic review of housing interventions found that moving people from higher to lower poverty neighbourhoods (deconcentrating poverty) may provide promising health outcomes, although more field evaluation is needed [93]. Such findings have implications for housing policies (e.g., mixed-income developments, and the design and placement of high-rise density public housing). While a substantial part of responses to housing affordability issues for families need to occur beyond the community level, better monitoring of housing issues at the local level may signal potential interventions. Housing issues may be possible underlying problems that place considerable stress on families, including their health and wellbeing. A report by the Australian Institute of Family Studies (AIFS) [75] highlights that local practitioners should include housing tenure and quality in routine assessments of their clients, and help clients explore ways to improve their living conditions (e.g., referrals to appropriate services, advocate to real estate agents and landlords for repairs to property structural deficiencies) [94]. Moreover, design (type), distribution (dispersion) of public housing, and revitalisation of poor quality public housing [93] may help ameliorate stigma associated with living in public housing.

This research was a secondary analysis of a larger project in which housing was only one subdomain of interest. Future research could include more purpose-built questions on housing issues affecting families with young children and taking a longitudinal perspective. For example, this may mean measuring the likely out-movers (displacement) from gentrification processes (e.g., tracking people moving in and out of the local area), and considering that processes of voluntary movement continue to impact family household decisions.

The communities in this study are not generalisable to more ‘advantaged’ communities. The issues of scope and generalisability are a critical consideration for studies of this nature. While we explored consistent housing factors that differ between ‘better than expected’ and ‘as expected’ local communities, our findings also re-iterate that neighbourhood effects and early childhood development cannot be generalised across all communities or all groups within communities. The intricacies of different community contexts limit generalisability and there are often more differences within, rather than between, neighbourhoods [5]. Notwithstanding disadvantage, neighbourhoods may also vary in terms of risk factors (e.g., crime rates, neighbourhood safety) and protective factors (e.g., social capital, collective efficacy) but these were not explored in detail in this paper. Thus, future studies should not only consider fit-for-purpose housing measures linked to early childhood development outcomes [7], but also measures of the surrounding neighbourhood built (e.g., green space [93], public transport) and social (e.g., social networks) environments. Family processes and socio-demographics can impact on the resources and opportunities available to children beyond area-level effects and should be included in models. In addition, quantitative data used in this study were sourced for a relatively small number of local communities, which represents challenges in the representativeness of quantitative results and ability to conduct further statistical modelling. Future studies should collect more objective measures of individual housing units.

Nevertheless, our qualitative findings re-emphasise that families who experience ‘double disadvantage’, being not only who you are, but also where you live, compounds the level of disadvantage experienced. Children from the most disadvantaged families living in a disadvantaged area (concentrated poverty) were particularly vulnerable to their local housing and neighbourhood contexts. In contrast, people who have a relatively higher socio-economic position living in a disadvantaged area may have the resources to change living conditions and/or travel beyond their neighbourhood to access employment, better services and destinations [95].

## 5. Conclusions

Better housing affordability, housing tenure and lower-density public housing were housing characteristics that emerged as points of difference for disadvantaged local communities where children had relatively better early childhood development outcomes. These all have the potential to be important, modifiable and policy sensitive levers for reducing early childhood development inequities. The mixed methods of this study provided a rich source of local information that could be triangulated and has implications for the current policy interest in place-based initiatives. Thoughtful design and dispersion of public housing, and providing affordable housing stock (rentals or home ownership) for families with young children, while delivering better neighbourhood built environments (e.g., green spaces, public transport) may be an important step in providing the conditions for reducing inequities in child health and development.

## Figures and Tables

**Table 1:** Housing measures of interest in the Kids in Communities Study.

	Factor/ Theme	Description of Housing Measure
1	Housing (includes all types of housing themes below)	Perceptions of housing in the community ^1^
2	Housing affordability	Perceptions of relationships between household income and spending on housing ^1,^* Proportion of households in the bottom 40% of equivalised disposable household income distribution spending more than 30% of gross household income on housing costs ^2^ [75]
3	Housing tenure	Perceptions of home ownership in the community ^1,^* Proportion of home ownership in the community ^2^
4	Public housing	Perceptions of public housing in the community ^1,^* Proportion of public housing in the community ^2^
5	Housing density	Perceptions of housing density in the community ^1,^* Objective residential density (number of dwellings/residential land area) and proportion of high-rise (three or more storeys) vs. low-rise ^2^

^1^ Perception as collected in qualitative interviews and focus groups; ^2^ Objective measure as collected in quantitative data; * Emerged from semi-structured qualitative findings, i.e., not directly asked of participants.

**Table 2:** Qualitative and quantitative housing findings for the matched-disadvantaged community pairs.

Theme/Factor	Hypothesis for Factor/Theme Differentiating Disadvantaged Communities Doing ‘Better Than Expected’ vs. ‘As Expected’ on ECD (OnDis Is Reference Group)	Hypothesis was Supported, Refuted, or Made No Difference to ECD in Disadvantaged Community Pair ^^^							Summary (≥4 Community Pairs ^^^)
		1	2	3	4	5	6	7	
Housing affordability	Housing perceived as more desirable in Off ^+,^*	~	✓	✓	✓	✓	~	✕	✓ (4 of 7)
	Housing stress: <40% of lower income households expend >30% of their income on housing costs in Off ^+,#^	~	~	~	~	✓	~	✓	~ (5 of 7)
	Housing affordability is perceived negatively because less disadvantaged families move into the area and displace more disadvantaged families in Off ^+,^*	~	✓	✓	✓	✓	~	✓	✓ (5 of 7)
Housing tenure	Lower proportion of renters (vs. home owners) in Off ^+,#^	✕	✓	✓	✓	✓	✓	✓	✓ (6 of 7)
Public housing	Perceived less public housing availability in Off ^+,^*	~	✓	✓	✓	✓	✓	✕	✓ (5 of 7)
	Lower proportion of public renters in Off ^+,#^	✓	✓	✓	~	✓	✓	~	✓ (5 of 7)
Housing density (linked to public housing)	Perceived less higher density housing (this refers to number of storeys) in Off ^+,^*	~	✓	✓	✓	✓	✓	✕	✓ (5 of 7)
	Lower proportion of higher density housing (3 or more storeys) in Off ^+^ (*not necessarily public housing*) ^#^	~	✓	~	~	✓	✓	✓	✓ (4 of 7)
Housing type (linked to public housing)	Perceived more separate house public housing in Off ^+,^*	~	✓	✓	✓	✓	✓	✕	✓ (5 of 7)
	Higher proportion of separate housing in Off ^+^ (*not necessarily public housing*) ^#^	~	~	~	~	✓	✓	✓	~ (4 of 7)
	Lower proportion of townhouses/apartments in Off ^+^ (*not necessarily public housing*) ^#^	~	✓	~	~	✓	✓	✓	✓ (4 of 7)

ECD: Early childhood development; * Perception; ^#^ Objective; Off ^+^: disadvantaged local community with ‘better than expected’ early childhood development outcomes; OnDis: disadvantaged local community with ‘expected’ early childhood development outcomes; ^^^ Disadvantaged community pair: Neighbouring Off ^+^ and OnDis local community matched on socio-economic disadvantage (Numbers 1–7 are the seven matched-disadvantaged pairs); ✓: Finding supports direction of hypothesis; ✕: Finding supports opposite direction of hypothesis; ~: Finding does not differentiate on- and off-diagonal local community.
